# Schizandrin A induces non‐small cell lung cancer apoptosis by suppressing the epidermal growth factor receptor activation

**DOI:** 10.1002/cam4.6942

**Published:** 2024-02-20

**Authors:** Linhai Zhu, Yanye Wang, Xuhua Huang, Xide Liu, Bo Ye, Yi He, Haojie Yu, Wang Lv, Luming Wang, Jian Hu

**Affiliations:** ^1^ Department of Thoracic Surgery, The First Affiliated Hospital Zhejiang University School of Medicine Hangzhou China; ^2^ Department of Arthropathy Zhejiang University of Traditional Chinese Medicine Affiliated Integrated Chinese and Western Medicine Hospital Hangzhou China; ^3^ Department of Thoracic Surgery Hangzhou Red Cross Hospital Hangzhou China; ^4^ State Key Laboratory of Subtropical Silviculture Zhejiang A&F University Hangzhou China; ^5^ State Key Laboratory of Chemical Engineering, College of Chemical and Biological Engineering Zhejiang University Hangzhou China; ^6^ Key Laboratory of Clinical Evaluation Technology for Medical device of Zhejiang Province Hangzhou China

**Keywords:** apoptosis, cell cycle, EGFR, non‐small cell lung cancer, Schizandrin A

## Abstract

**Objective:**

The purpose of this study is to explore the biological mechanism of Schizandrin A (SchA) inducing non‐small cell lung cancer (NSCLC) apoptosis.

**Methods:**

The reverse molecular docking tool “Swiss Target Prediction” was used to predict the targets of SchA. Protein–protein interaction analysis was performed on potential targets using the String database. Functional enrichment analyses of potential targets were performed with Gene Ontology and Kyoto Encyclopedia of Genes and Genomes. The conformation of SchA binding to target was simulated by chemical‐protein interactomics and molecular docking. The effect of SchA on the expression and phosphorylation level of EGFR was detected by Western blot. Lipofectamine 3000 and EGFR plasmids were used to overexpress EGFR. Apoptosis was tested with Annexin V‐FITC and propidium iodide staining, and cell cycle was detected by propidium iodide staining.

**Results:**

The “Swiss Target Prediction” database predicted 112 and 111 targets based on the 2D and 3D structures of SchA, respectively, of which kinases accounted for the most, accounting for 24%. Protein interaction network analyses showed that molecular targets such as ERBB family and SRC were at the center of the network. Functional enrichment analyses indicated that ERBB‐related signaling pathways were enriched. Compound‐protein interactomics and molecular docking revealed that SchA could bind to the ATP‐active pocket of the EGFR tyrosine kinase domain. Laboratory results showed that SchA inhibited the phosphorylation of EGFR. Insulin could counteract the cytotoxic effect of SchA. EGFR overexpression and excess EGF or IGF‐1 had limited impacts on the cytotoxicity of SchA.

**Conclusions:**

Network pharmacology analyses suggested that ERBB family members may be the targets of SchA. SchA can inhibit NSCLC at least in part by inhibiting EGFR phosphorylation, and activating the EGFR bypass can neutralize the cytotoxicity of SchA.

## 1 INTRODUCTION

World Health Organization's International Agency for Research on Cancer (WHO‐IARC) reported in the “Global Cancer Statistics 2020” the lung cancer deaths (1.8 million cases) ranked first in the total global cancer deaths (9.96 million cases).^1^ Non‐small cell lung cancer (NSCLC), including lung squamous cell carcinoma, lung adenocarcinoma, and large cell carcinoma, is the most common type of lung cancer, accounting for approximately 80%–85% of all lung cancers.^2^ In recent years, great progress has been made in the treatment of NSCLC. With the clinical application of targeted drugs against EGFR, ALK, ROS1, MET, NTRK mutations, and immune checkpoint inhibitors against PD‐L1/PD‐1, the overall survival of advanced NSCLC patients has been significantly prolonged.^3^, ^4^ However, after a period of targeted therapy or immunotherapy, cancer cells eventually develop drug resistance and progress, which is the most important cause of cancer death.^5^, ^6^ Drug resistance has become an urgent medical problem to be solved, and exploring new effective therapeutic drugs is crucial to overcome drug resistance and prolong the survival of lung cancer patients.

Purifying natural products into monomeric compounds and studying their effects on cancer is one of the ways to explore potential anticancer drugs. Schizandrin A (SchA), also known as Deoxyschizandrin, is one of the most biologically active lignans isolated from the fruit of medicinal plant Schizandra chinensis.^7^ Xian et al. reported that SchA combined with gefitinib could inhibit cell growth and promote apoptosis in gefitinib‐resistant NSCLC cells.^8^ A recent study found that for NSCLC cells, SchA induces G1/S arrest at low concentrations and G2/M arrest and apoptosis at high concentrations.^9^ SchA may be a potential drug for the treatment of NSCLC, but the molecular mechanism of its effect is still unclear.

Network pharmacology is an emerging discipline of “drug‐target‐disease” relationship network based on systems biology and multidirectional pharmacology, which is used to systematically and comprehensively observe the impact of drugs on disease networks.^10^ Accumulating researches have proved that network pharmacology is a feasible systematic approach to uncovering the molecular mechanisms by which drugs exert physiological effects or to searching for small molecule drugs that may have therapeutic effects.^11^, ^12^ In this study, we used network pharmacology combined with laboratory methods to explore the possible molecular mechanism of the anti‐tumor effect of SchA.

## 2 MATERIALS AND METHODS

### 2.1 Cell culture and treatment

NSCLC cell lines A549 (EGFR‐wild type) and H1975 (EGFR‐L858R/T790M) were purchased from The Cell Bank of Type Culture of The Chinese Academy of Sciences. The cells were cultured in RPMI1640 medium supplemented with 10% FBS (PAN Biotech #P30‐3302, Bavaria, Germany) in 37°C in a humidified atmosphere with 5% CO_2_. SchA, with a purity of more than 98%, was purchased from Chengdu Must Bio‐Technology Co. Ltd (#19092908, Chengdu, Sichuan, China). Epidermal growth factor (EGF) was purchased from R&D Systems (#236‐EG, R&D Systems, MN, USA). Insulin‐like growth factor 1 (IGF1) was purchased from SinoBiological (#105980HNAE, SinoBiological, Beijing, China). Insulin was purchased from Sigma (#I2643, Sigma, MO, USA). In EGF activation assays, the cells were starved for 24 h in FBS‐free medium and then pre‐treated with SchA 50 μM for 4 h, and then EGF 100 ng/mL was used to stimulate the cells at planned time points of 0, 5, 10, 30, 60 min.

### 2.2 Target prediction and PPI network construction

Reverse docking is a technique for finding potential targets for a given small active molecule by computational methods.^13^ The query molecule SchA was submitted to a web‐based reverse docking service “SwissTargetPrediction,” with the aim of predicting the most probable protein targets of SchA (http://www.swisstargetprediction.ch/). The predictions provided by “SwissTargetPrediction” were based on a combination of molecular 2D and 3D structural similarity with a library of 376,342 compounds known to be experimentally active on an extended set of 3068 macromolecular targets.^14^, ^15^

The predicted target proteins were used for protein–protein interaction (PPI) analysis through the String database (https://string‐db.org/, version 11.0).^16^ The intermolecular connections in the PPI network were counted and ranked by R software (version 4.0).

### 2.3 Pathway enrichment analysis

Gene ontology (GO) analysis, including three components of cell components (CC), biological processes (BP), and molecular functions (MF), is a comprehensive resource for functional annotation of genes and gene products.^17^ GO analysis and MF enrichment analysis were performed. Kyoto Encyclopedia of Genes and Genomes (KEGG) is a practical database resource that can perform enrichment analysis of molecular data sets to obtain advanced functions of biological systems.^18^ The potential target genes for SchA were mapped to GO and KEGG databases for functional enrichment analysis using clusterProfiler package in R software.^19^

### 2.4 Molecular docking and MD simulation

Chemical‐protein interactome (CPI) is an interaction information matrix of a panel of drugs across multiple protein molecules constructed using a variety of strategies, including chemical structure comparison, molecular docking, text‐mining, etc.^20^ DRAR‐CPI is a web server that attempts to explore unexpected drug‐protein interactions by mining CPI, showing the accuracy of bindings through docking score and normalized value Z'‐score.^21^ The smaller the docking score and Z'‐score, the more accurate the small molecule drug binds to the target protein.^22^ The molecular file of SchA structure was uploaded to the DRAR‐CPI server (https://cpi.bio‐x.cn/drar/). Gefitinib, as a positive control molecule, was also input into DRAR‐CPI for a query.

Molecular docking was conducted by AutoDock Vina (version 4.2) to predict the noncovalent binding of SchA and EGFR protein.^23^ Based on the PDB ID provided by the DRAR‐CPI, the target protein EGFR molecular conformation was downloaded from the PDB database (https://www.rcsb.org/). The conformation of EGFR L858R and L858R/T790M mutants was also obtained from the PDB database. Gefitinib and osimertinib, as positive controls, were also involved in molecular docking. The target protein and ligands were prepared using the AutoDockTools software (version 1.5). The complex of ligand and protein binding was visualized using PyMol software (version 2.6).

Molecular dynamic (MD) simulations were performed using the GROMACS software (version 2021.6). The EGFR mutants (L858R and L858R/T790M) and SchA were described by AMBER and GAFF2 force field parameters. The restrained electrostatic potential (RESP) charge was used to describe the atomic charge of SchA. The excess charge of the system was neutralized with Na + or Cl− ions. The steepest gradient method was used to minimize the energy of the EGFR‐SchA complex in 3000 steps under vacuum conditions to eliminate the unreasonable overlap between atoms. After the complex was solvated by adding SPC water to the box, the energy of the solvated system was minimized again under the position‐limited complex conditions, and then the solvent was relaxed under NVT and NPT traces, respectively. Finally, the position limitation of the complex was removed, and the whole system was simulated for 100 ns under the NPT trace. In the MD simulation, periodic boundary conditions were used in all three directions, and the LINCS algorithm was used to constrain the bond lengths of all hydrogen‐containing chemical bonds (C‐H, O‐H) with a time step of 2.0 fs. The temperature was maintained at 298.15 K by means of a V‐rescale thermostat with a coupling time of 0.5 ps, and the pressure was controlled at 1 bar by a Parrinello–Rahman thermostat with a coupling time of 2.0 ps. The particle grid Ewald (PME) method was used to calculate the long‐range electrostatic interaction, and the short‐range electrostatic and VDW interaction was calculated using the cut‐off value of 1.2 nm. Trajectory analysis and visualization were performed using GROMACS software and VMD software (version 1.9.3).

### 2.5 Western blot analysis

After being exposed to SchA for 24 hs, A549 and H1975 cells were harvested by trypsinization and pelleted by centrifugation. The pellets were lysed in RIPA lysis buffer (#P0013B, Beyotime Co., Shanghai, China) supplemented with protease inhibitor cocktail (#05892791001, Roche, Germany) and phosphatase inhibitor cocktail (#524629, Millipore, MA, USA). Protein concentrations were determined using the BCA protein assay kit (#PC0020, Solarbio, Beijing, China). Protein samples were separated by SDS‐PAGE and then electro‐transferred to PVDF membranes (#IPVH00010, Millipore, Ireland). After blocking with 5% BSA in Tris‐buffered saline (TBS), the membranes were incubated with specific primary antibodies overnight at 4°C. Primary antibodies against pEGFR (Tyr1068) (#3777) and EGFR (#4267) were obtained from Cell Signaling Technology (MA, USA). Primary antibody against β‐actin was purchased from Sigma‐Aldrich (#A1978, St. Louis, MO, USA). Depending on the molecular size of the protein and the protein marker (#26617, Thermo Fisher Scientific Co., MA, USA), we horizontally cut the PVDF membrane into bands containing the target protein for incubation. The protein bands were washed with TBS‐Tween 20 and then incubated with HRP‐conjugated secondary antibodies (anti‐rabbit and anti‐mouse, CST, MA, USA) at room temperature for 1 hour. After being washed three times with TBS‐Tween 20, protein bands were visualized using ECL chemiluminescence reagents (#BL523B, Biosharp, Hefei, China).

### 2.6 Cell cycle and cell apoptosis analyses by flow cytometry

To verify the effects on cell cycle distribution and the percentage of apoptotic cells by SchA, cells were examined by flow cytometry. For cell cycle analysis, A549 and H1975 cells were incubated with SchA or DMSO for 24 hours, and then fixed in pre‐chilled 70% ethanol overnight. Fixed cells were washed with PBS and stained with propidium iodide (PI)/RNase staining buffer (#550825, BD, San Jose, CA, USA). Subsequently, cell cycle distribution was measured on flow cytometer (Beckman Coulter, Inc., California, USA).

Cell apoptosis samples were prepared using Annexin V‐FITC/PI Apoptosis Detection Kit I (#556547, BD, San Jose, CA, USA) and quantified by flow cytometer (Beckman Coulter, Inc., California, USA). The flow cytometric results were visualized with FlowJo 10 (BD, San Jose, CA, USA).

### 2.7 Cell transfection

The pLVX‐EGFR‐FLAG‐puro (NM_005228.5) plasmid was obtained from Heyin Biosciences Co., Ltd (Hangzhou, China). EGFR plasmid were transfected into A549 and H1975 cells using Lipofectamine 3000 (Invitrogen, CA, USA) according to the manufacture's protocol. At 24 h post‐transfection, the cells were treated with SchA.

### 2.8 Statistical analysis

The experiments except immunoblot assays were replicated at least three times. All values were expressed as mean ± standard deviation (SD). The two‐tailed Student's *t* test or two‐way ANOVA with Bonferroni's multiple comparisons test was used to evaluate the quantitative data for statistical significance. *p* < 0.05 was considered statistically significant.

## 3 RESULTS

### 3.1 SchA target prediction and PPI network analysis

**FIGURE 1:**
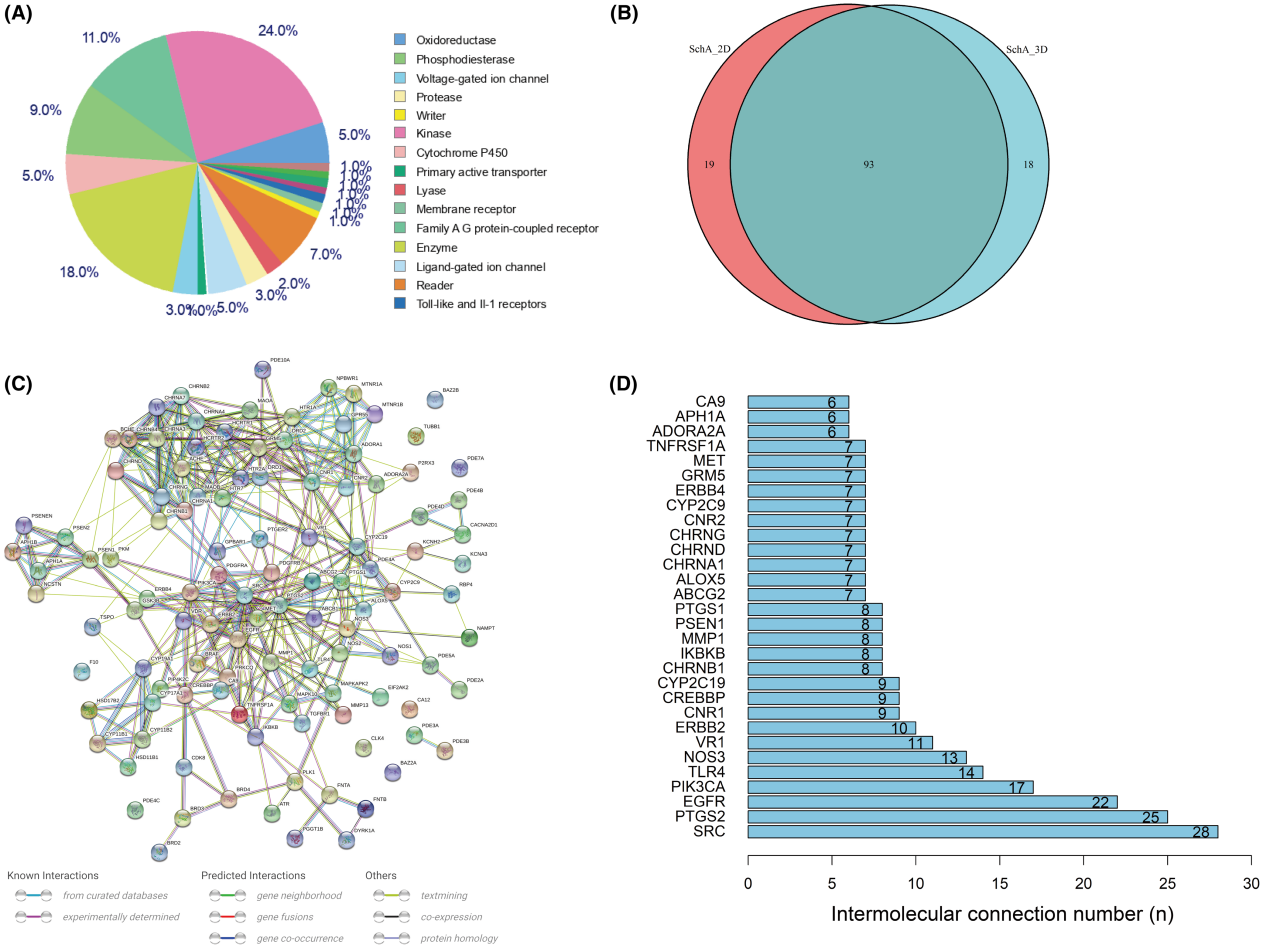
The structure of SchA and its predicted targets. (A) The 2D structural forms of SchA. (B) The 3D structural forms of SchA. (C) The classification of SchA's predicted targets. (D) The intersection of the prediction results based on 2D and 3D forms of SchA. (E) The PPI network based on 93 target molecules using the String tool. (F) The top 30 nodes with the most connections in PPI network. PPI, protein–protein interaction; SchA, Schizandrin A.

The 2D and 3D structural forms of SchA (PubChem CID: 43595) were downloaded from PubChem (https://pubchem.ncbi.nlm.nih.gov/) and used as query molecules to search possible protein targets in the “SwissTargetPrediction” web tool. A total of 112 protein molecules were retrieved using SchA in 2D form, and 111 protein molecules were retrieved in 3D form. Among these predicted targets, kinases accounted for 24%, followed by enzymes (18%), G protein‐coupled receptor family (11%), and phosphodiesterase (9%) (Figure 1A). These classification results suggested that SchA may function as a kinase. The intersection of the prediction results based on 2D and 3D forms was 93 molecules (Figure 1B), which were considered as candidate target molecules for further analysis.

The PPI network based on 93 target molecules was constructed by the String tool (Figure 1C). The nodes that had more connections with other nodes in the network were considered hub nodes. The top 30 nodes with the most connections were shown in Figure 1D. Although SRC (non‐receptor tyrosine kinase) ranked first, when EGFR, ErbB2, and ErbB4 was considered as a family, the EGFR tyrosine kinase family that acted as homo‐ or heterodimer had more connections.^24^, ^25^ Therefore, these three molecules of the EGFR family could be counted as the hub molecules in the PPI network. Compared with ErbB3, EGFR, ErbB2, and ErbB4 all have a complete tyrosine kinase domain, so it is reasonable to believe that SchA acts on the tyrosine kinase domain of the EGFR family.

### 3.2 Biological pathway enrichment analysis

**FIGURE 2:**
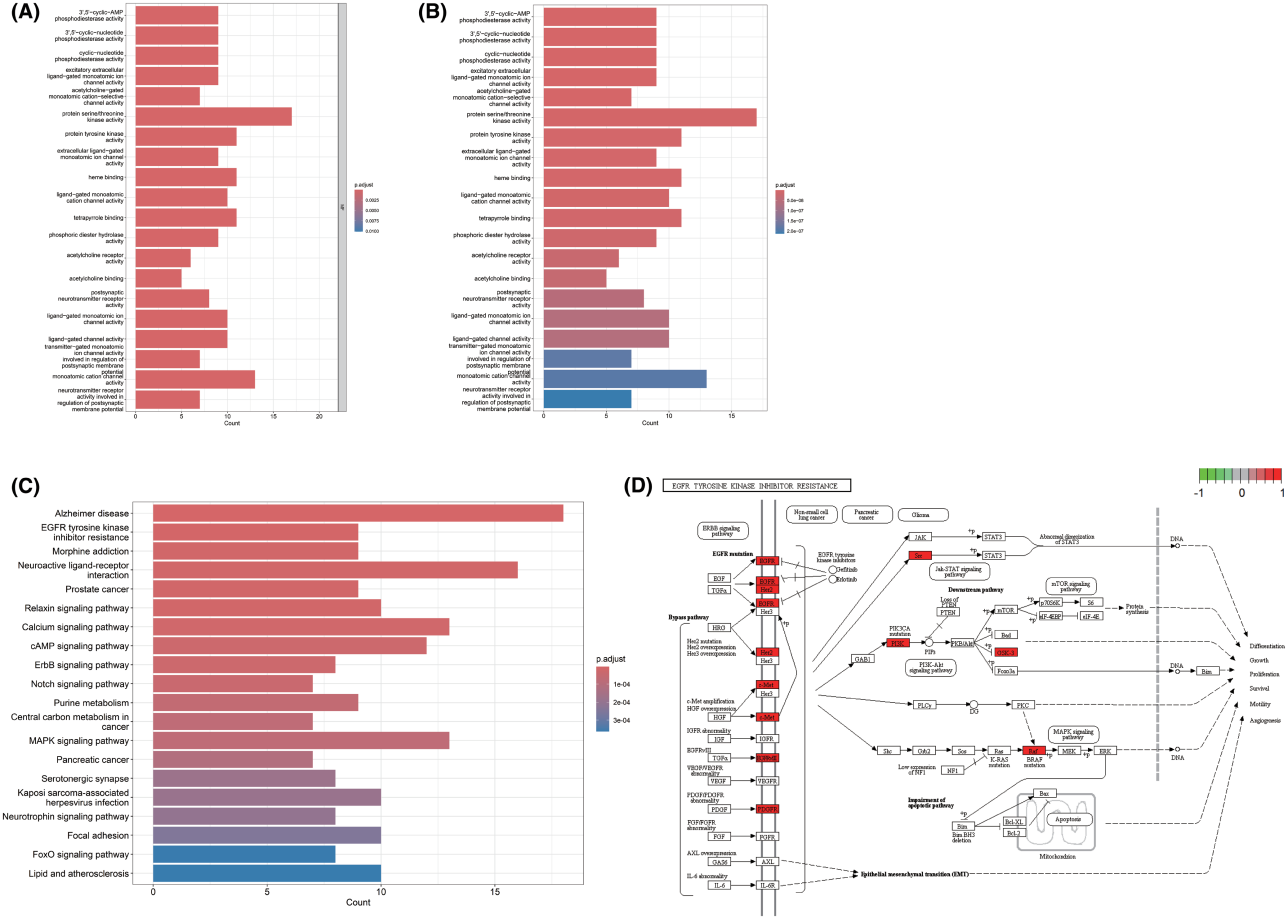
Functional enrichment analysis of predicted target molecules for SchA. (A) Histogram of the first 20 items of the GO analysis MF module. (B) Histogram of the first 20 items of the MF enrichment analysis. (C) Histogram of the first 20 items of the KEGG enrichment analysis. (D) The “EGFR tyrosine kinase inhibitor resistance signaling pathway” and related molecules (marked in red) provided by KEGG. GO, gene ontology; KEGG, Kyoto Encyclopedia of Genes and Genomes; MF, molecular functions; SchA, Schizandrin A.

The items of GO analysis results were ranked according to statistical difference. The first 20 items of MF module were displayed in histograms (Figure 2A). “Protein tyrosine kinase activity” related to 10 genes such as CLK4, DYRK1A, EGFR, EIF2AK2, ERBB2, ERBB4, MET, PDGFRA, PDGFRB, and SRC was enriched in MF module (*p* < 0.001). MF enrichment analysis was further performed to evaluate the molecular function of SchA effects (Figure 2B). “Protein tyrosine kinase activity” related to ten genes such as CLK4, DYRK1A, EGFR, EIF2AK2, ERBB2, ERBB4, MET, PDGFRA, PDGFRB, PKM, and SRC was enriched (*p* < 0.001).

KEGG enrichment analysis also provided insights for exploring biological pathways related to SchA targets (Figure 2C). The “EGFR tyrosine kinase inhibitor resistance” (*p* < 0.001) and “ERBB signaling pathway” (*p* < 0.001) were significantly enriched in the KEGG analysis (Figure 2C). Since the abnormal activation of the EGFR signaling pathway is one of the hallmarks of lung cancer, we took the EGFR signaling pathway as the focus of further research. The “EGFR tyrosine kinase inhibitor resistance” signaling pathway and related molecules (marked in red) provided by KEGG were shown in Figure 2D.

### 3.3 Molecular interaction modeling by molecular docking and MD simulation

The molecular structures of SchA and gefitinib were uploaded to the DRAR‐CPI web server separately, and the results showed that all these two small molecule drugs could bind EGFR protein molecule (PDB ID: 1M17, Table 1). According to the normalized Z'‐score, SchA (Z'‐score: −0.608) was more likely to bind to EGFR than gefitinib (Z'‐score: −0.374) (Table 1).

**TABLE 1:** The chemical‐EGFR interaction analysis based on DRAR‐CPI.

PDB ID	Target protein	Small molecule	Docking score	Z'‐score
1M17	EGFR	SchA	−42.5621	−0.608181
1M17	EGFR	Gefitinib	−54.3804	−0.373699

Abbreviations: DRAR‐CPI, drug repositioning potential and adverse drug reactions via the chemical‐protein interactome; PDB, Protein data bank; SchA, Schizandrin A.

**FIGURE 3:**
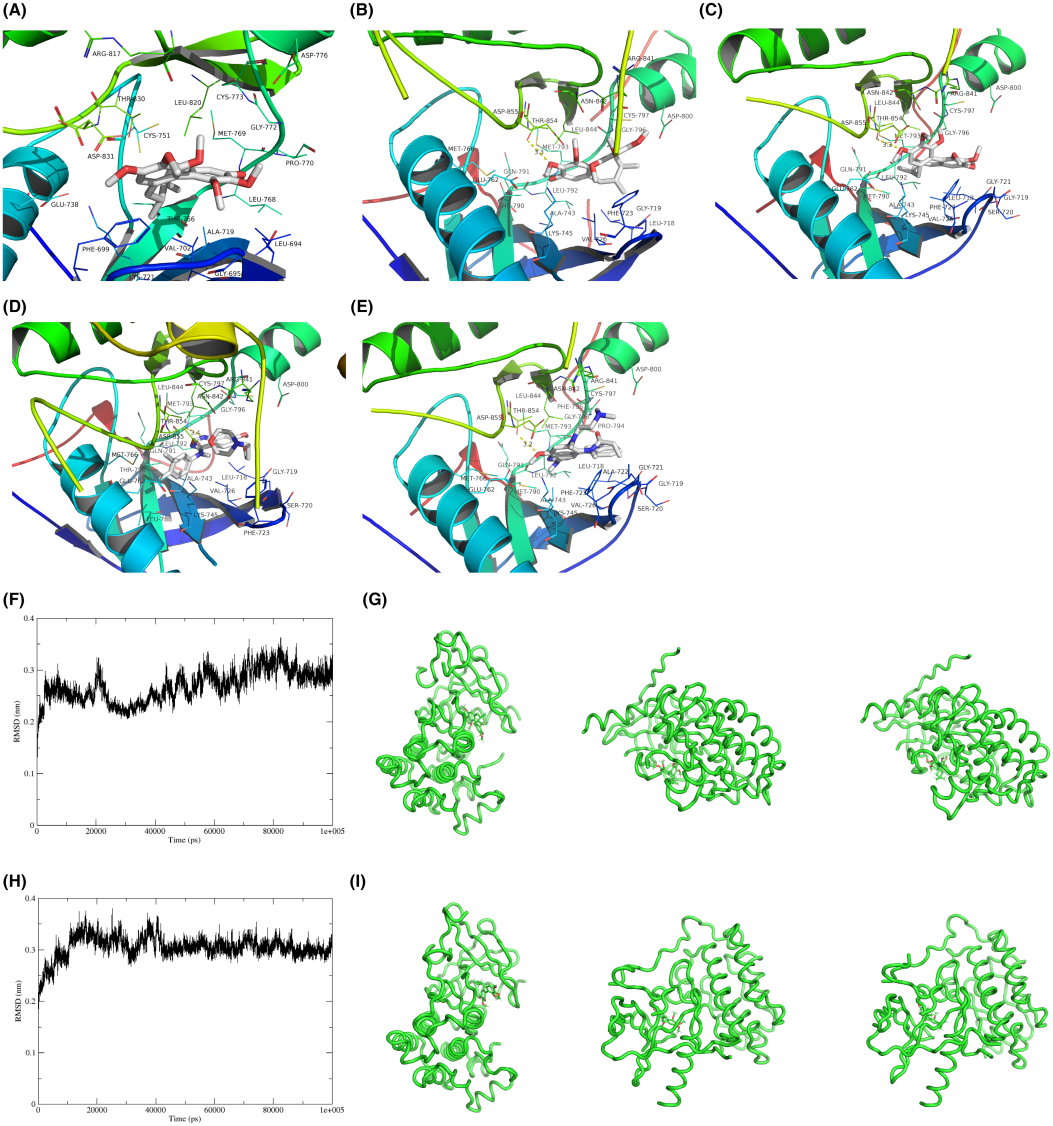
The interactions of EGFR molecules with SchA, gefitinib or osimertinib. (A) The interaction between SchA and EGFR (1M17) molecule. The binding between SchA and EGFR WT was supported by the π‐π stacking interaction provided by PHE‐699. (B) The interaction between SchA and EGFR mutant L858R (PDB ID 4LQM) molecule. The hydrogen bond provided by THR‐854 and the π‐π stacking interaction provided by PHE‐723 promoted the binding affinity in SchA‐EGFR L858R complex. (C) The interaction between SchA and EGFR mutant L858R/T790M (PDB ID 4RJ7) molecule. The hydrogen bond provided by THR‐854 and the π‐π stacking interaction provided by PHE‐723 promoted the binding affinity in SchA‐EGFR L858R/T790M complex. (D) The interaction between gefitinib and EGFR mutant L858R (PDB ID 4LQM) molecule. The combination of gefitinib and EGFR L858R is supported by the hydrogen bonding provided by MET‐793 and the π‐π stacking interaction provided by PHE‐723. (E) The interaction between osimertinib and EGFR mutant L858R/T790M (PDB ID 4RJ7) molecule. The combination of osimertinib and EGFR mutant L858R/T790M is supported by the hydrogen bonding provided by THR‐854 and the π‐π stacking interaction provided by PHE‐723. (F) MD simulation of SchA‐EGFR L858R complex. RMSD of SchA‐EGFR L858R complex. (G) MD simulation snapshot of the SchA‐EGFR L858R complex. (H) MD simulation of SchA‐EGFR L858R/T790M complex. RMSD of SchA‐EGFR L858R/T790M complex. (I) MD simulation snapshot of the SchA‐EGFR L858R/T790M complex. MD, molecular dynamic; RMSD, root mean square deviation; SchA, Schizandrin A.

The interaction between SchA and EGFR (1M17) molecule was also verified by AutoDock Vina, and the lowest binding affinity was −7.7 kcal/mol (Figure 3A, Table 2). In addition to overexpression, EGFR activating mutations also play an essential role in tumorigenesis, development and drug resistance.^26^ To detect the effect of SchA on EGFR mutations, the interaction of SchA with EGFR mutants L858R (PDB ID 4LQM, Figure 3B) and L858R/T790M (PDB ID 4RJ7, Figure 3C) were also tested. At the same time, the interaction of gefitinib with EGFR mutant L858R (PDB ID 4LQM, Figure 3D) and osimertinib with EGFR mutant L858R/T790M (PDB ID 4RJ7, Figure 3E) were used as positive controls. The binding affinity of SchA to EGFR L858R and L858R/T790M were −7.6 kcal/mol and −7.5 kcal/mol, respectively, which were slightly lower than that of gefitinib‐EGFR L858R (−8.0 kcal/mol) and osimertinib‐EGFR L858R/T790M (−8.3 kcal/mol) (Table 2).

**TABLE 2:** The binding site and the lowest binding energy by molecular docking.

PDB ID	Target protein	Small molecule	Center (x, y, z)	Binding affinity
1M17	EGFR WT	SchA	21.697, 0.303, 52.093	−7.7 kcal/mol
4LQM	EGFR L858R	SchA	−52.671, −2.43, −23.967	−7.6 kcal/mol
4RJ7	EGFR T790M/L858R	SchA	−51.545, −1.386, −20.813	−7.5 kcal/mol
4LQM	EGFR L858R	Gefitinib	−52.671, −2.43, −23.967	−8.0 kcal/mol
4RJ7	EGFR T790M/L858R	Osimertinib	−51.545, −1.386, −20.813	−8.3 kcal/mol

Abbreviations: PDB, protein data bank; SchA, Schizandrin A.

Visualization of the binding pattern revealed that SchA and gefitinib were most likely to bind to EGFR near the ATP‐binding cleft of the kinase domain. The binding between SchA and EGFR WT was supported by the π–π stacking interaction provided by PHE‐699 (Figure 3A). The hydrogen bond provided by THR‐854 and the π–π stacking interaction provided by PHE‐723 promoted the binding affinity in SchA‐EGFR L858R complex (Figure 3B) and SchA‐EGFR L858R/T790M (Figure 3C). The combination of gefitinib and EGFR L858R might be supported by the hydrogen bonding provided by MET‐793 and the π–π stacking interaction provided by PHE‐723 (Figure 3D), and the combination of osimertinib and EGFR L858R/T790M might be supported by the hydrogen bonding provided by THR‐854 and the π–π stacking interaction provided by PHE‐723 (Figure 3E).

We used Gromacs to perform 100 ns MD simulations of the SchA and EGFR mutant (L858R and L858R/T790M) complexes, and used Root Mean Square Deviation (RMSD) to explore the stability of the binding of SchA and EGFR mutants (L858R and L858R/T790M). RMSD fluctuations and simulation snapshots of the SchA‐EGFR mutant (L858R) complex were shown in Figure 3F,G. RMSD fluctuations and simulation snapshots of the SchA‐EGFR mutant (L858R/T790M) complex were shown in Figure 3H,I. It can be seen from the Figure 3F,H that the RMSD values of the SchA‐EGFR mutant (L858R) complex and the SchA‐EGFR mutant (L858R/T790M) complex fluctuated slightly, indicating that the binding of SchA to the EGFR mutant (L858R) and the EGFR mutant (L858R/T790M) was relatively stable.

### 3.4 SchA inhibited the phosphorylation of EGFR

**FIGURE 4:**
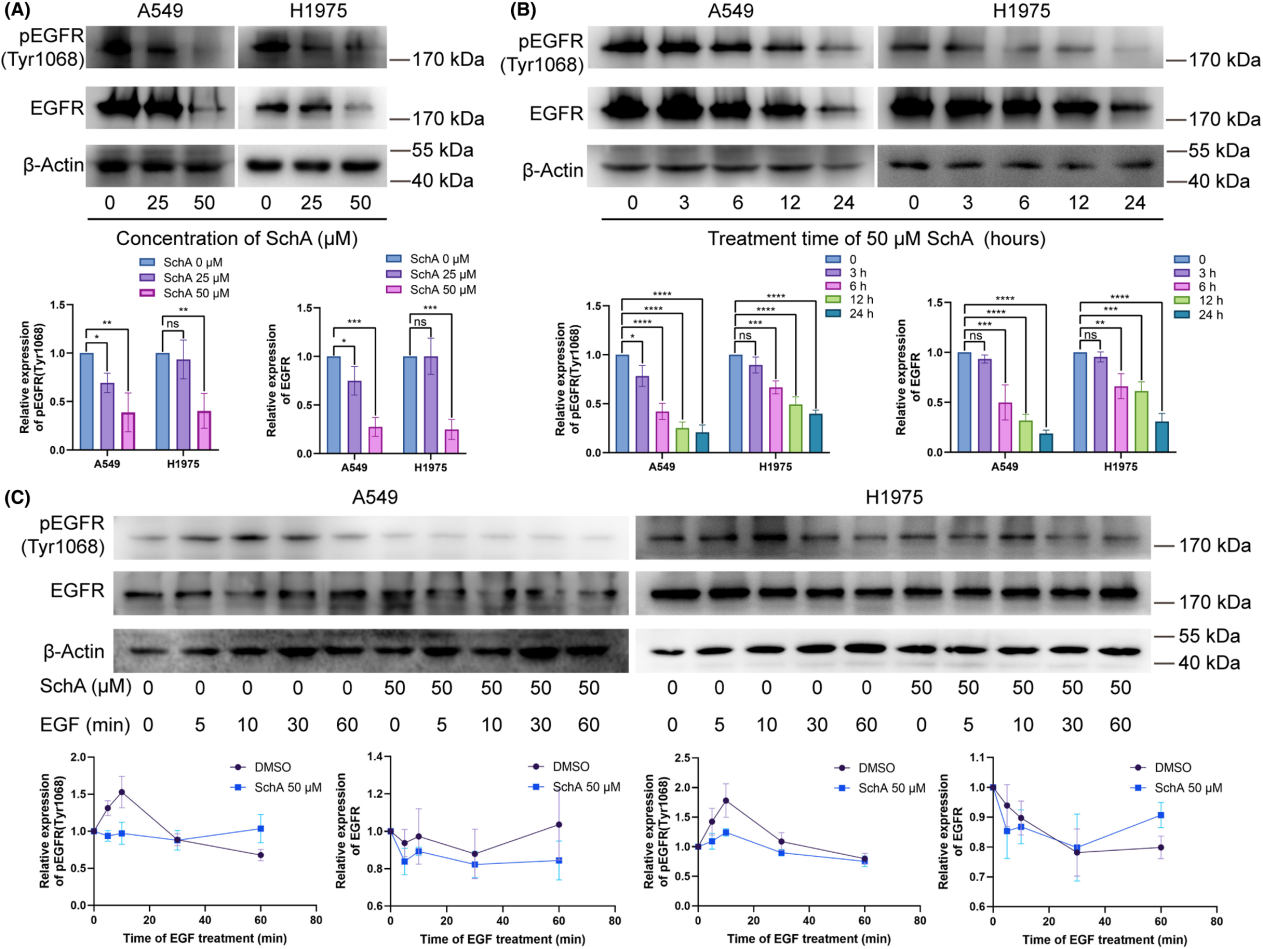
SchA inhibited the phosphorylation of EGFR. (A) SchA resulted in the inhibition of EGFR phosphorylation (Tyr 1068) in a dose‐dependent manner in A549 and H1975 cells. (B) SchA resulted in the inhibition of EGFR phosphorylation (Tyr 1068) in a time‐dependent manner in A549 and H1975 cells. (C) EGF‐induced EGFR phosphorylation (Tyr 1068) was inhibited by SchA pre‐treatment 4 h. MW, molecular weight; SchA, Schizandrin A. ns, *p* ≥ 0.05; *p < 0.05; **p < 0.01; ***p < 0.001; ****p < 0.0001.

To investigate whether SchA can inhibit EGFR kinase activity, we examined the effect of SchA on EGFR phosphorylation. As shown in Figure 4A,B, exposure of A549 and H1975 cells to SchA resulted in the inhibition of EGFR phosphorylation (Tyr 1068) in a dose‐ and time‐dependent manner. The total EGFR also showed a trend of consumption after SchA treatment.

In addition, we tested the effect of SchA on EGF‐induced EGFR activation. After 4 hours of SchA pre‐treatment, EGF‐induced phosphorylation (Tyr 1068) of EGFR were inhibited (Figure 4C). These results indicated that SchA could inhibit EGFR phosphorylation (Tyr 1068).

### 3.5 Activating the EGFR pathway with overexpressing EGFR or adding EGF could not reduce SchA‐induced cytotoxicity

**FIGURE 5:**
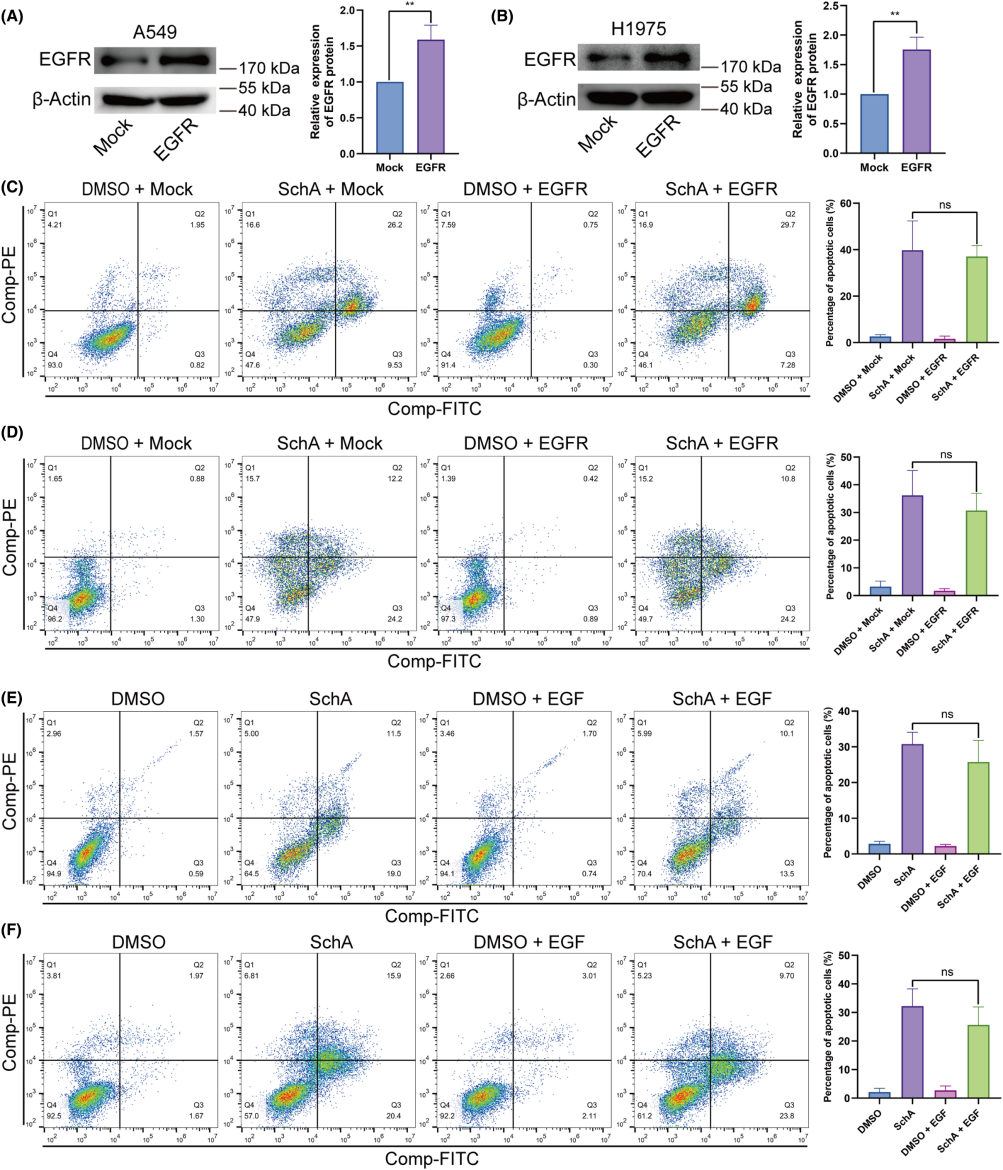
The effects of EGFR overexpression and addition of EGF on SchA‐induced apoptosis. (A) The level of EGFR expression in A549 cells was measured by western blot. (B) The level of EGFR expression in H1975 cells was measured by western blot. (C) The elevated EGFR expression level had no significant effect on SchA‐induced apoptosis in A549 cells. (D) The elevated EGFR expression level had no significant effect on SchA‐induced apoptosis in H1975 cells. (E) EGF stimulation had no significant impact on SchA‐induced apoptosis in A549 cells. (F) EGF stimulation had no significant impact on SchA‐induced apoptosis in H1975 cells. SchA, Schizandrin A.

To further clarify the role of EGFR expression in SchA‐mediated cytotoxicity, A549 and H1975 cells were transfected with LVX‐EGFR‐FLAG‐puro plasmid to overexpress EGFR. Then the cytotoxicity of SchA in EGFR overexpressing cells was evaluated. After transfected with LVX‐EGFR‐FLAG‐puro plasmid, the expression of EGFR was substantially increased in A549 (Figure 5A) and H1975 (Figure 5B) cells. By examining the apoptosis of SchA‐treated A549 (Figure 5C) and H1975 (Figure 5D) cells with high EGFR expression, we found that the elevated EGFR expression level had no significant effect on SchA‐induced apoptosis. Next, we used EGF to activate the EGFR signaling pathway and then observed the cytotoxic effect of SchA. We also found that EGF stimulation of A549 (Figure 5E) and H1975 (Figure 5F) cells had no significant impact on SchA‐induced apoptosis. This result suggests that SchA exerts a cytotoxic effect by inhibiting EGFR activation.

### 3.6 Activating the EGFR bypass pathways reduced SchA‐induced cytotoxicity partially

**FIGURE 6:**
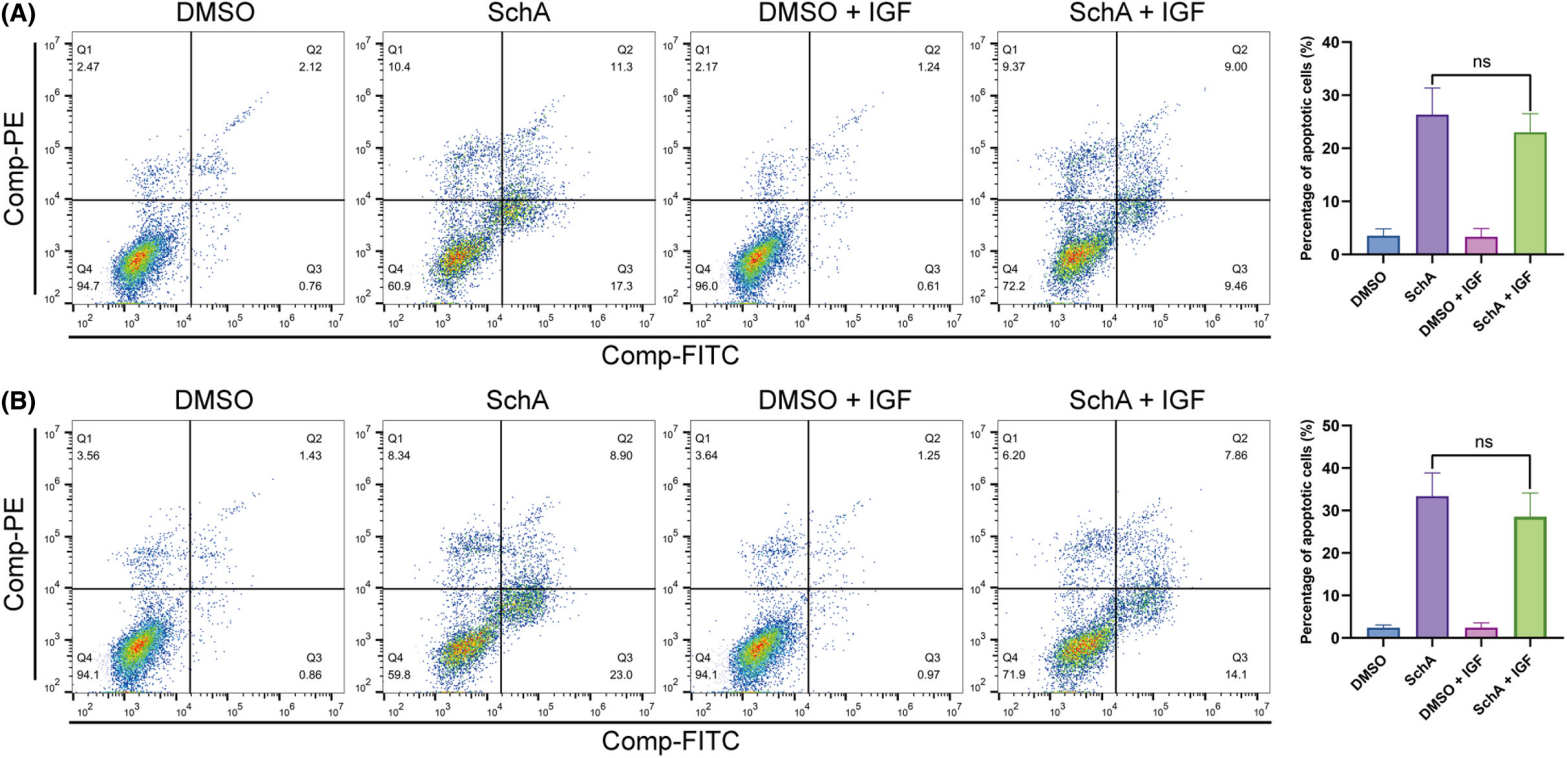
The effects of IGF stimulation on SchA‐induced apoptosis. (A) IGF stimulation had no significant impact on SchA‐induced apoptosis in A549 cells. (B) IGF stimulation had no significant impact on SchA‐induced apoptosis in H1975 cells. SchA, Schizandrin A.

**FIGURE 7:**
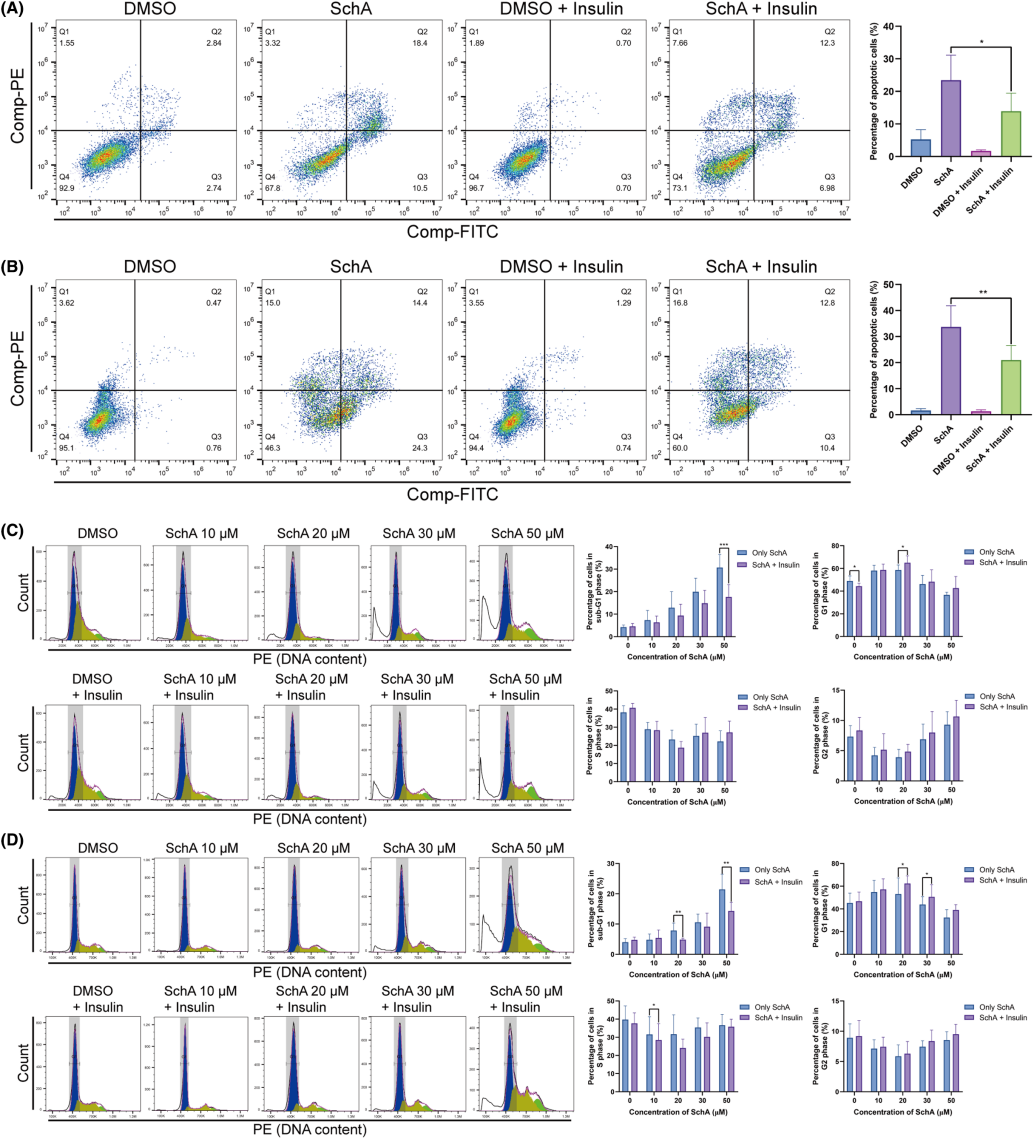
The effects of insulin stimulation on SchA‐induced cytotoxicity. (A) Insulin stimulation can significantly attenuate SchA‐induced apoptosis of A549 cells. (B) Insulin stimulation can significantly attenuate SchA‐induced apoptosis of H1975 cells. (C) For A549 cells, insulin reduced the proportion of SchA‐induced sub‐G1 phase cells and increased the proportion of cells stagnant in the G1 phase. (D) For H1975 cells, insulin reduced the proportion of SchA‐induced sub‐G1 phase cells and increased the proportion of cells stagnant in the G1 phase. SchA, Schizandrin A.

The IGF signaling pathway and the insulin signaling pathway are essential EGFR bypass pathways and share the downstream growth factor signaling pathway with EGFR. In this study, we added IGF or insulin to activate the downstream growth factor signaling pathway. Then we observed the effect of IGF or insulin on SchA cytotoxicity. The addition of IGF to SchA‐treated cells had no apparent impact on SchA‐induced apoptosis (Figure 6). Furthermore, we stimulated SchA‐treated cells with insulin and found that insulin attenuated the apoptosis induced by SchA (Figure 7A,B). Cell cycle detection experiments also showed that insulin reduced the proportion of cells in the sub‐G1 phase caused by SchA (Figure 7C,D).

## 4 DISCUSSION

In recent years, SchA has garnered attention for its potential anti‐tumor effects, with studies highlighting diverse mechanisms of action. Lee et al. demonstrated SchA's ability to induce cell cycle arrest in ovarian cancer cells by inhibiting cyclin E expression and AKT activation.^27^ Similarly, investigations on triple‐negative breast cancer revealed SchA's role in inducing cell cycle arrest and apoptosis through modulation of Wnt and endoplasmic reticulum stress signaling pathways.^28^ Notably, SchA has also been implicated in colorectal cancer, where it induced cell cycle arrest and apoptosis by inhibiting heat shock factor 1.^29^ Furthermore, SchA has shown promise in overcoming doxorubicin resistance in breast cancer cells by inhibiting P63 and Stat3 phosphorylation.^30^

Extending beyond these findings, our study employed network pharmacology to identify potential target molecules of SchA, revealing kinases and enzymes as the main targets. The subsequent protein–protein interaction (PPI) network analysis emphasized the centrality of molecules with tyrosine kinase activity, including the ERBB family, SRC, and MET. ERBB‐related signaling pathways were enriched, suggesting a potential mechanism through which SchA may influence lung cancer cells. ERBB‐related signaling pathways play a crucial role in lung cancer, and targeted therapies directed against these pathways have shown significant clinical impact. Drugs such as gefitinib and osimertinib, targeting EGFR, are effective in treating NSCLC patients with EGFR activating mutations.^31^ Trastuzumab and other HER2‐targeted therapies are being explored for lung cancer patients with HER2 mutation.^32^

Experimental validation revealed SchA's capacity to inhibit EGFR activation. Molecular docking studies demonstrated SchA's affinity for the EGFR tyrosine kinase domain, comparable to gefitinib and osimertinib, indicating a potential role in inhibiting EGFR kinase activity. However, it is noteworthy that SchA exhibited higher binding affinity to wild‐type EGFR compared to mutant EGFR, suggesting potential side effects as an anti‐tumor drug. Further in vivo experiments should be conducted to enhance the credibility of these results.

Interestingly, enhancing the EGFR signaling pathway had limited impact on SchA's cytotoxic effects, possibly due to the extensive occupation of the EGFR tyrosine kinase active site by SchA. Additionally, the addition of IGF1 had minimal effect, suggesting that the IGF1R signaling pathway may not be a primary pathway in NSCLC. Probably because the IGF1R signaling pathway was not the main signaling pathway in NSCLC.^33^ However, excess insulin counteracted SchA's cytotoxicity by activating the insulin receptor signaling pathway, an EGFR bypass pathway.

The potential inhibitory targets underscored SchA's potential inhibitory effects on a variety of kinases, including EGFR, HER2, HER4, SRC, and MET. Our study primarily confirms SchA's inhibitory effect on EGFR, emphasizing the need for further investigation into its impact on other potential target molecules.

## 5 CONCLUSION

In this study, the network pharmacology method was used to explore the possible targets and biological pathways of SchA. Combined with laboratory studies, it was proved that SchA could inhibit NSCLC at least in part by inhibiting the EGFR tyrosine kinase.

## AUTHOR CONTRIBUTIONS

**Linhai Zhu:** Conceptualization (lead); writing – review and editing (lead). **Yanye Wang:** Data curation (lead); writing – original draft (lead). **Xuhua Huang:** Data curation (lead); visualization (lead). **Xide Liu:** Data curation (equal); funding acquisition (supporting); supervision (supporting). **Bo Ye:** Conceptualization (equal); methodology (equal); resources (supporting). **Yi He:** Investigation (equal); supervision (equal). **Haojie Yu:** Project administration (equal); software (equal). **Wang Lv:** Software (supporting); supervision (supporting); writing – review and editing (equal). **Luming Wang:** Methodology (equal); resources (supporting); validation (equal). **Jian Hu:** Funding acquisition (lead); writing – review and editing (lead).

## FUNDING INFORMATION

This research was supported by the Zhejiang Provincial Traditional Chinese Medicine (Integrated Traditional Chinese and Western Medicine) Key Discipline (grant numbers 2017‐XK‐A33), the National Key Research and Development Program (grant numbers 2017YFC0113500), the Zhejiang Province Major Science and Technology Special Program Project (grant numbers 2020C03058), the Zhejiang Province Lung Tumor Diagnosis and Treatment Technology Research Supported by the Center (grant numbers JBZX‐202007) and the Zhejiang Medicine and Health General Research Program (grant numbers 2019KY069).

## CONFLICT OF INTEREST STATEMENT

The authors declare no conflict of interest. The funders had no role in the design of the study; in the collection, analyses, or interpretation of data; in the writing of the manuscript, or in the decision to publish the results.

## Data Availability

The 2D and 3D structural forms of SchA (PubChem CID: 43595) could be downloaded from PubChem at https://pubchem.ncbi.nlm.nih.gov/. The web‐based reverse docking service “SwissTargetPrediction” could be assessed at http://www.swisstargetprediction.ch/. Protein–protein interaction analysis could be assessed in the String database at https://string‐db.org/, version 11.0. Chemical‐protein interactome could be analyzed through DRAR‐CPI web server at https://cpi.bio‐x.cn/drar/. All other data on which the results and conclusions of this study are based, are available from the corresponding author on reasonable request.
